# Scoliosis in adolescents reduces the risk of eating disorders

**DOI:** 10.1186/1748-7161-7-S1-O7

**Published:** 2012-01-27

**Authors:** F Zaina, S Donzelli, M Lusini, M Togoingar, V Negro, S Negrini

**Affiliations:** 1 ISICO Milan, Italy; 2 Siena University, Italy; 3 Università Cattolica del Sacro Cuore di Milano, Italy

## Background and purpose

A recent study suggests a correlation between idiopathic scoliosis in adolescence and eating disorders [1]. Nevertheless, this did not correspond to our clinical experience in this same population. The aim of this study was to verify the correlation between scoliosis and eating disorders in adolescence.

## Materials and methods

Design: cross-sectional study.

Population: 187 consecutive adolescent girls with idiopathic scoliosis (mean Cobb angle 26°, range 11-73°, age 15.2±2.5; 24% juveniles, 76% adolescent type); 93 school girl controls (age 14.9±1.0).

All the subjects answered the Italian validated questionnaire EAT-26 about eating habitude in order to retrieve eating disorders. BMI was calculated for all subjects and compared to reference data.

Statistical Analysis: chi-square test and ANOVA.

## Results

Only 3 (1.6%; IC95 -0.2/3.4%) subject in the scoliosis group showed EAT-26 scores suggestive for eating disorders versus 7 (7.5%; IC95% 2.2/12.9%) in the school population; the difference was statistically significant (p<0.05). The odds ratio of eating disorders in adolescents with scoliosis is 0.2 (IC95 –1.18/1.58). BMI was slightly lower (p<0.05) for scoliosis patients (19±0.2) that for school girls (21±0.3).

## Conclusions

EAT-26 is recognised among the most valid questionnaires for eating disorders and has been widely applied in various countries. Applying it, we found a lower incidence of eating disorders in female scoliosis patients than in the general population (both our own controls and Italian reference values). This contrast with some expert opinions and a recent study performed in Italy. The low BMI already reported in the literature as typical of scoliosis subjects is confirmed by our data.
